# Supplementary material for: Structural and Aggregation Features of a Human κ-Casein Fragment with Antitumor and Cell-Penetrating Properties
Source: Molecules. 2019 Aug 12;24(16):2919. doi: 10.3390/molecules24162919 (PMC6721048; doi:10.3390/molecules24162919)
Supplement: Supplementary file 1 [file molecules-24-02919-s001.pdf]

Structural and aggregation features of human milk κ-casein with antitumor and cell-penetrating properties

Olga Chinak[1], Andrey Shernyukov[2,3], Sergey Ovcherenko[2,3], Evgeniy Sviridov[2,3], Victor Golyshev[1], Alexandr Fomin[1], Inna Pyshnaya[1], Elena Kuligina[1], Vladimir Richter[1], Elena Bagryanskaya[2,3]

[1] Institute of Chemical Biology and Fundamental Medicine SB RAS, Novosibirsk, Russia
[2] N.N. Vorozhtsov Novosibirsk Institute of Organic Chemistry SB RAS, Novosibirsk, Russia,
[3] Novosibirsk State University, 630090, Novosibirsk, Pirogova, 2, Russian Federation

Electronic Supplementary Information

Table of contents

Fig. S1. Disorder prediction by DISOclust S2
Fig. S2. RL2 particle diameters in different pH and ionic strength. S3
Fig. S3. HSQC spectra of RL2 at pH 7 (left) and pH 3.5 (right) S4
Fig. S4. HSQC spectra of RL2 in H2O (left), 50% TFE (right) and their combination (bottom). S5
Fig. S5. HSQC spectra of [U-^{13}C,^{15}N]-RL2 at pH=3.9 (blue, Acetate buffer) and pH 3.5 (orange). S6
Table 1 Chemical shifts of RL2 at pH 3.5 S7
Table 2 The values of ^{3}J(HN-Hα) at pH 3.5 S9
Table 3 Relaxation data and SSP results for RL2 at pH 3.5 S11
Table 4 Chemical shifts of RL2 at pH=3.9 (Acetate buffer) S14
Fig. S6. Comparison of protein <T_1> values S17
Fig. S7. Fragment ^{1}H NMR spectrum of RL2 before and after TCEP addition. S18
Fig. S8. Gel filtration chromatograms showing the separation of RL2 dimer from the monomer. S19

RL2 sequence:

```
MNQKQPACHE   NDERPFYQKT   APYVPMYYVP   NSYPYYGTNL   YQRRPAIAIN
NPYVPRTYYA   NPAVVRPHAQ   IPQRQYLPNS   HPPTVVRRPN   LHPSFIAIPP
KKIQDKIIIP   TIGGSHHHHH   H
```

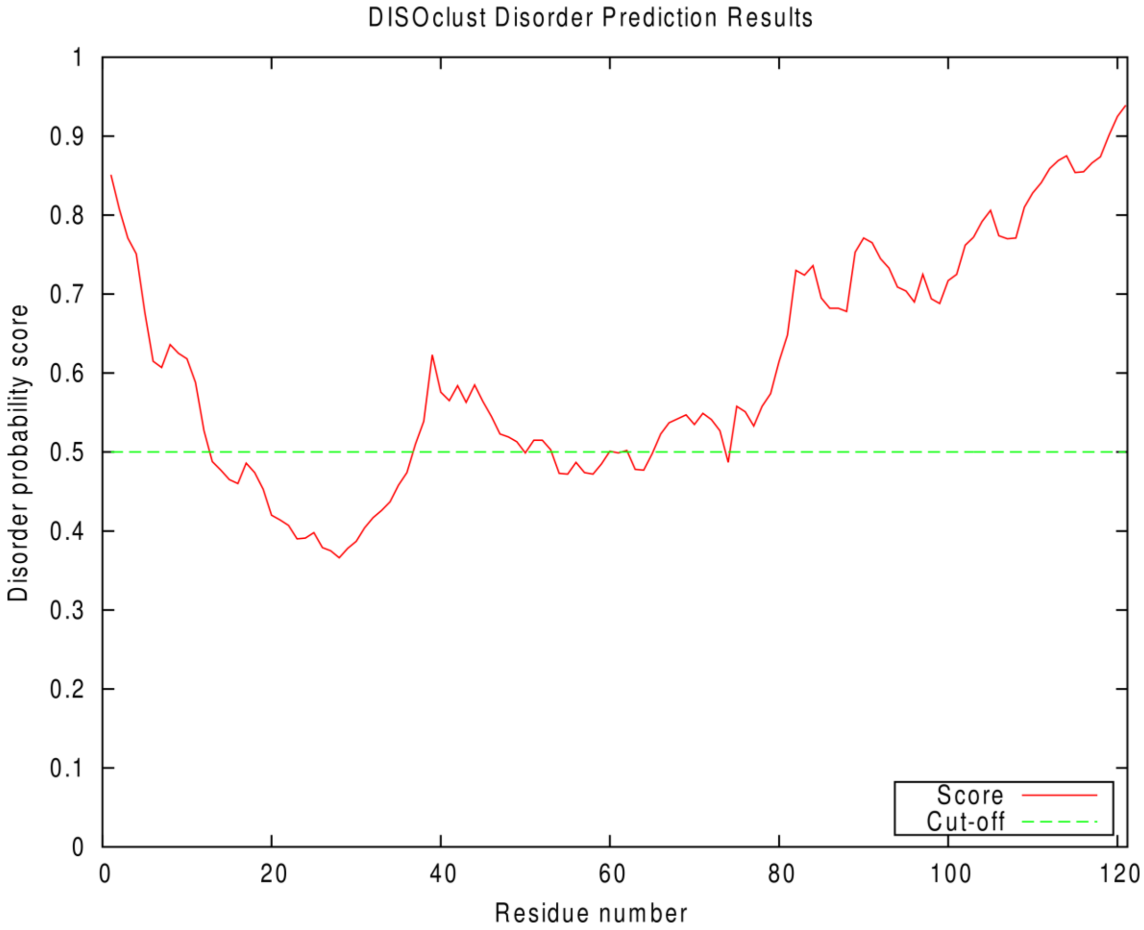

Fig. S1. Disorder prediction by DISOclust

Disorder prediction is carried out using DISOclust:
McGuffin, L. J. (2008) Intrinsic disorder prediction from the analysis of multiple protein fold recognition models. Bioinformatics, 24, 1789-1804

The results were obtained with the IntFOLD server:

McGuffin, L.J., Atkins, J., Salehe, B.R., Shuid, A.N. & Roche, D.B. (2015) IntFOLD: an integrated server for modelling protein structures and functions from amino acid sequences. Nucleic Acids Research, 43, W169-73. doi: 10.1093/nar/gkv236

Buenavista, M. T., Roche, D. B. & McGuffin, L. J. (2012) Improvement of 3D protein models using multiple templates guided by single-template model quality assessment. Bioinformatics, 28, 1851-1857. DOI: 10.1093/bioinformatics/bts292

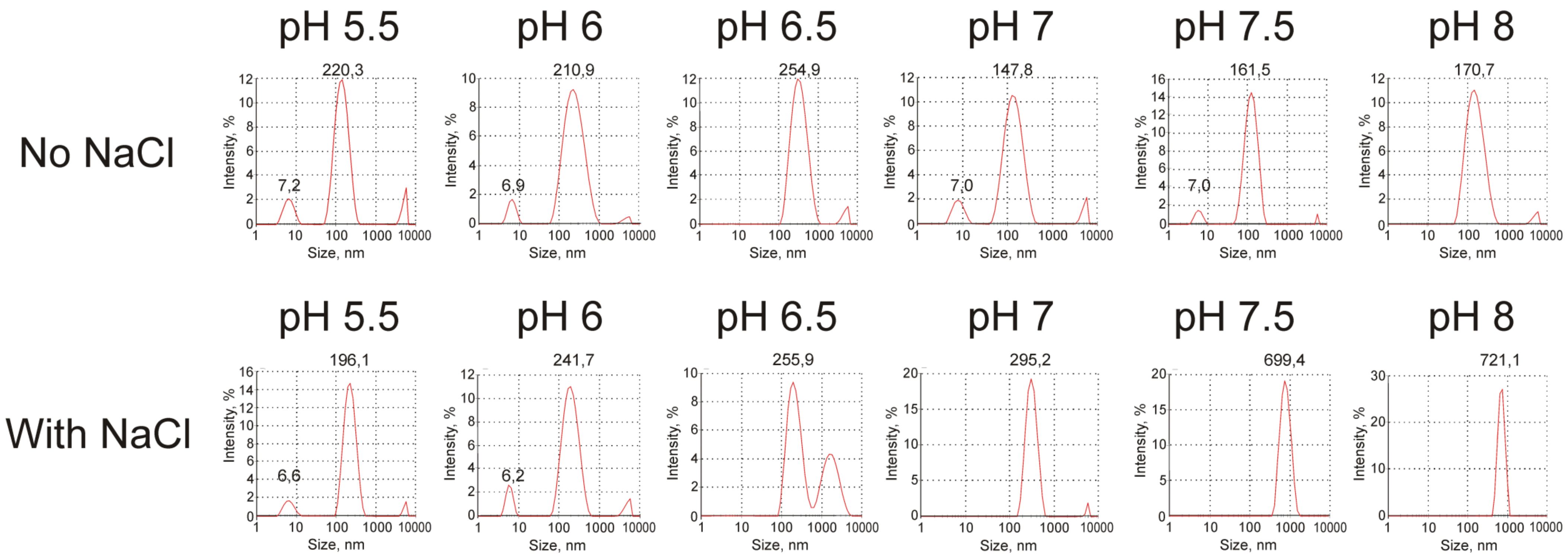

Fig. S2. RL2 particle diameters in different pH and ionic strength by DLS.

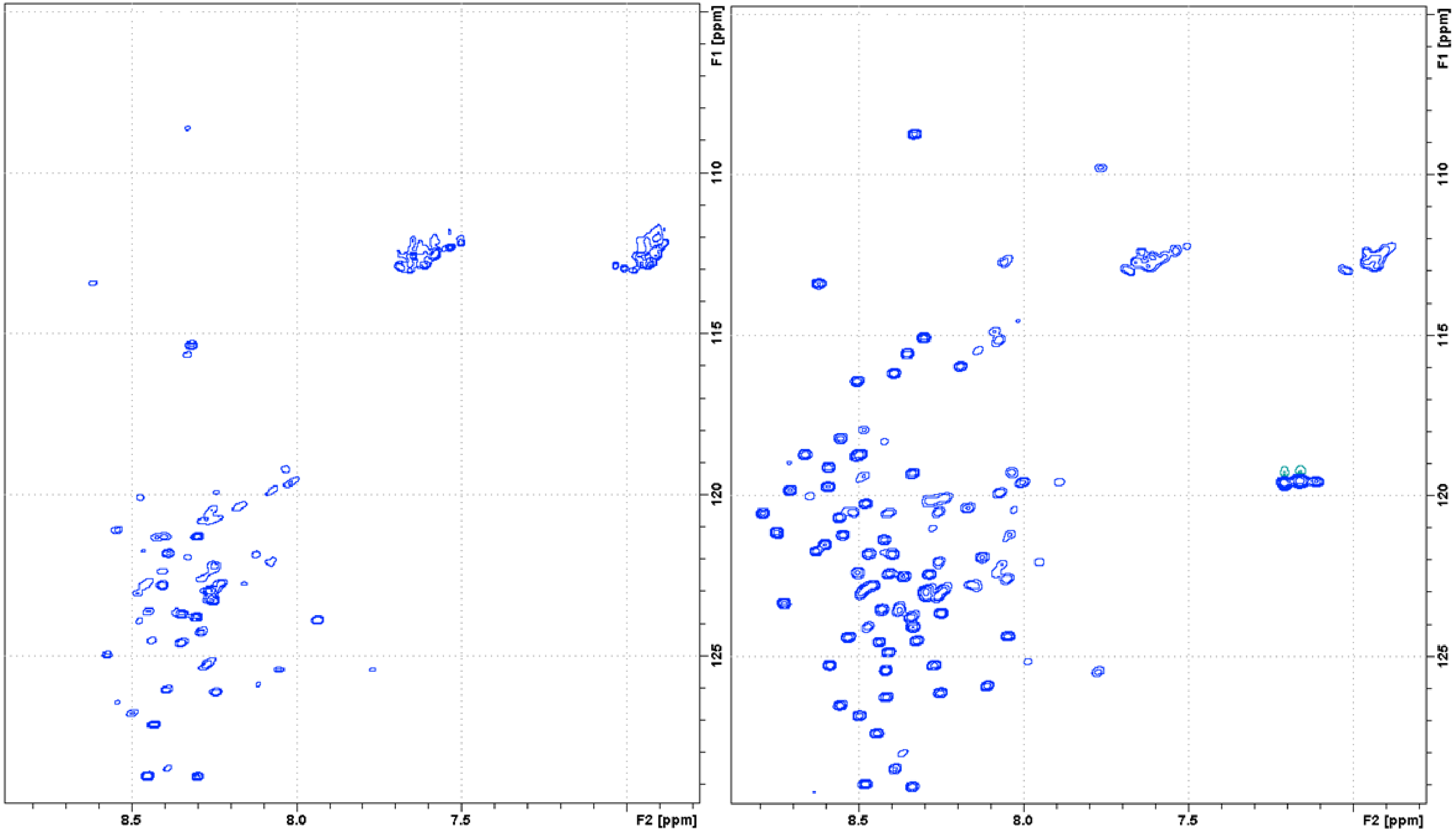

Fig. S3. HSQC spectra of RL2 at pH 7 (left) and pH 3.5 (right)

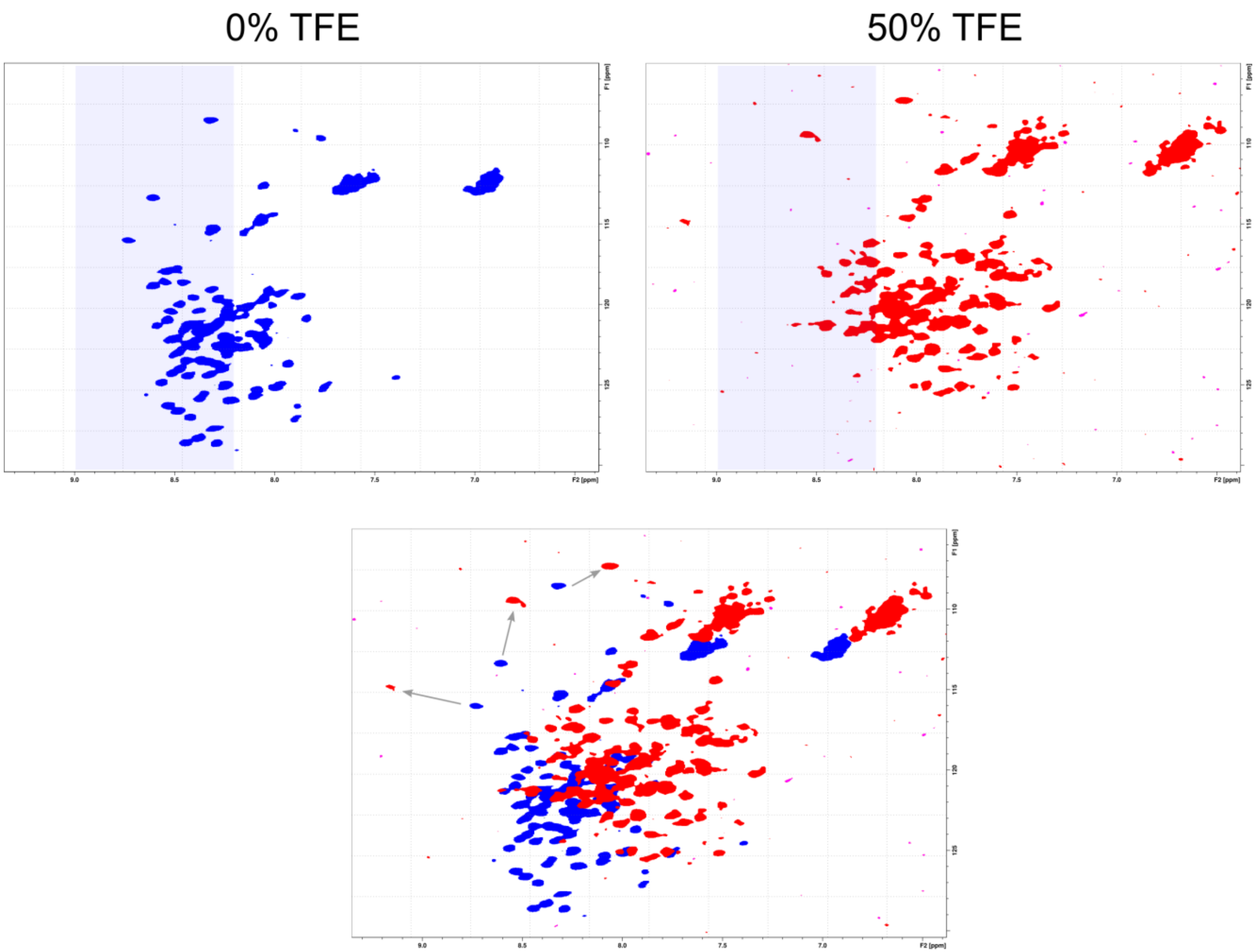

Fig. S4. HSQC spectra of RL2 in H_2O (left), 50% TFE (right) and their superimposition (bottom). Highlighted region is characteristic for random-coil shifts:

1) Steven P. Mielke1 and V.V. Krishnan. Characterization of protein secondary structure from NMR chemical shifts // Prog Nucl Magn Reson Spectrosc. 2009 Vol. 54(3-4). P. 141–165. doi:10.1016/j.pnmrs.2008.06.002.

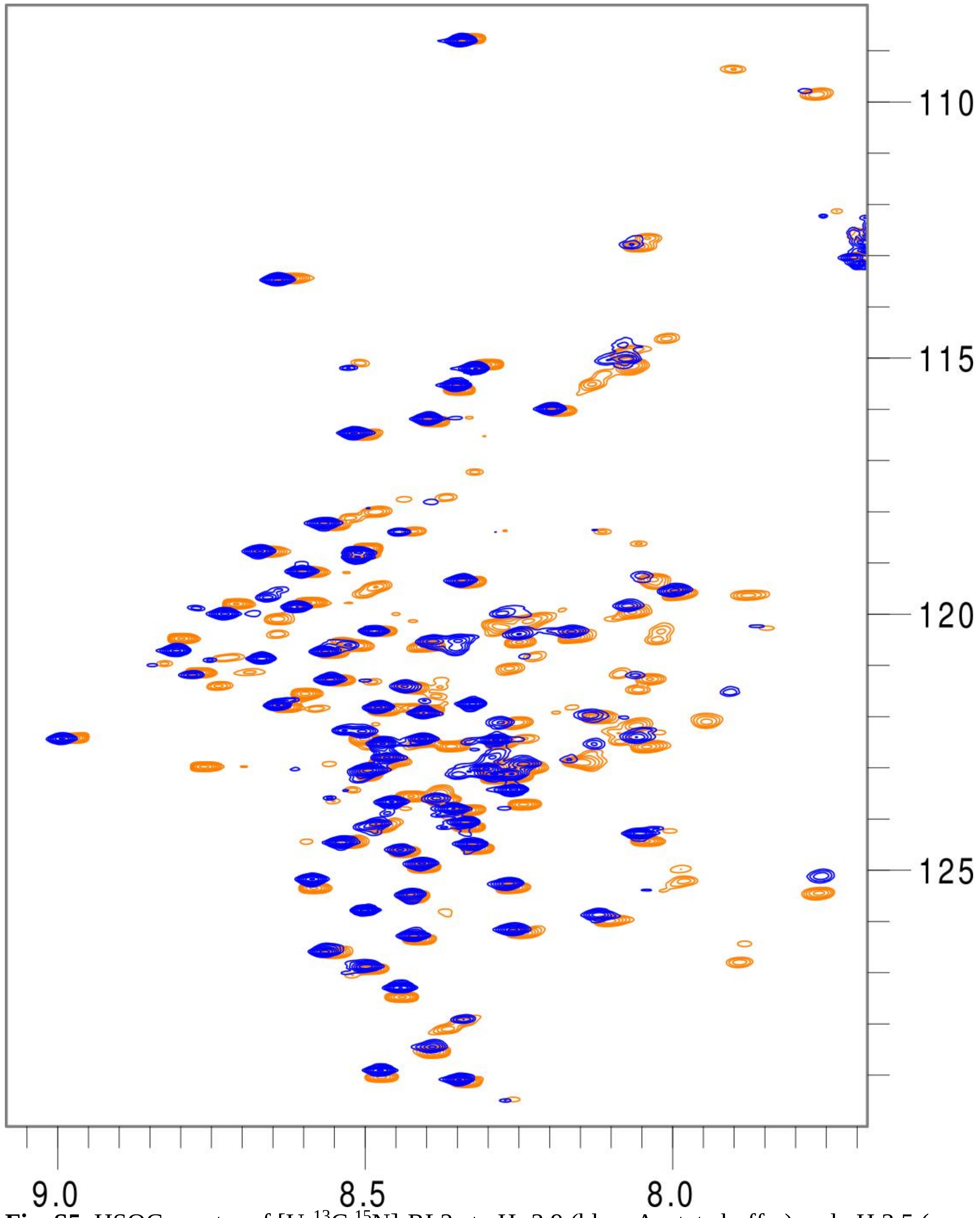

Fig. S5. HSQC spectra of [U-^{13}C,^{15}N]-RL2 at pH=3.9 (blue, Acetate buffer) and pH 3.5 (orange).

Table 1 Chemical shifts of BME-RL2 at pH 3.5

N		δH	δN	δH^{α}	δH^{β}	δH^{γ}	δH^{δ}	δC′	δC^{α}	δC^{β}
1	Met	-	-	4.15	-	-	-	172.05	55.05	32.61
2	Asn	8.98	122.42	4.77	-	-	-	174.75	-	-
3	Gln	8.63	121.82	4.34	-	-	-	-	55.83	29.59
4	Lys	8.49	123.10	4.30	-	-	-	176.41	56.41	32.88
5	Gln	8.48	122.92	4.63	-	-	-	173.93	53.55	28.94
6	Pro	-	-	4.40	-	-	-	176.49	63.06	31.99
7	Ala	8.47	124.16	4.31	-	-	-	177.61	52.48	19.08
8	Cys	8.42	118.34	4.64	-	-	-	174.54	55.38	41.09
9	His	8.74	121.40	4.74	-	-	-	174.05	55.20	28.79
10	Glu	8.42	121.87	4.36	-	-	-	175.64	56.05	28.73
11	Asn	8.65	120.15	4.70	-	-	-	175.01	53.42	38.65
12	Asp	8.49	119.46	4.72	-	-	-	174.96	52.98	37.86
13	Glu	8.29	120.25	4.36	-	-	-	175.69	55.86	28.52
14	Arg	8.29	123.04	-	-	-	-	-	-	-
16	Phe	-	-	-	-	-	-	175.19	57.93	39.44
17	Tyr	7.95	122.10	4.49	2.91, 2.91	-	-	175.06	57.58	38.96
18	Gln	8.14	122.89	4.24	-	-	-	175.27	55.38	29.83
19	Lys	8.38	123.51	4.26	-	-	-	176.65	56.79	32.93
20	Thr	8.13	115.51	4.30	-	1.22	-	173.80	61.55	69.81
21	Ala	8.37	128.08	4.58	1.38	-	-	175.36	50.62	18.10
30	Pro	-	-	4.25	-	-	-	176.60	63.21	31.97
31	Asn	8.48	118.06	4.56	2.77, 2.77	-	-	174.96	53.70	38.58
32	Ser	8.08	114.92	-	-	-	-	173.63	58.23	63.93
33	Tyr	8.16	122.89	4.73	-	-	-	174.09	55.89	38.40
36	Tyr	-	-	4.51	-	-	-	176.05	57.97	38.86
37	Gly	7.77	109.85	3.89, 3.89	-	-	-	174.08	45.35	-
38	Thr	8.06	112.81	4.35	-	1.22	-	174.51	61.83	69.78
39	Asn	8.52	120.59	4.68	2.78, 2.78	-	-	175.26	53.39	38.52
40	Leu	8.13	122.05	4.18	-	-	0.79, 0.79	177.17	55.86	42.17
41	Tyr	8.04	119.34	4.49	3.01, 3.01	-	-	175.71	58.00	38.32
42	Gln	8.04	121.29	4.25	-	-	-	175.39	55.89	29.49
43	Arg	8.26	122.12	4.27	-	-	-	175.91	56.06	30.62
45	Pro	-	-	-	-	-	-	176.41	63.07	32.00
46	Ala	8.44	124.65	4.29	1.37	-	-	177.66	52.50	19.06
47	Ile	8.07	119.95	4.14	-	-	-	175.75	60.95	38.85
48	Ala	8.39	128.53	4.36	1.37	-	-	177.41	52.35	19.20
49	Ile	8.16	120.45	4.13	-	-	-	175.86	61.13	38.83
50	Asn	8.50	122.46	4.69	2.74, 2.74	-	-	174.27	53.03	39.01
51	Asn	8.40	120.62	4.95	2.76, 2.76	-	-	173.29	51.20	-
55	Pro	-	-	-	-	-	-	176.76	62.98	32.03
56	Arg	8.42	121.42	4.31	-	-	-	176.37	56.29	30.80

57	Thr	8.07	115.14	4.25	4.06	1.08	-	173.81	61.69	69.74
58	Tyr	8.23	123.00	4.51	2.89, 2.89	-	-	174.94	57.98	39.16
59	Tyr	8.05	122.59	4.48	-	-	-	174.75	57.52	39.14
60	Ala	8.11	125.99	4.22	-	-	-	176.62	51.97	19.32
61	Asn	8.33	119.38	4.88	-	-	-	173.62	51.12	38.73
62	Pro	-	-	-	-	-	-	176.55	63.38	31.94
63	Ala	8.25	123.17	4.28	1.38	-	-	177.82	52.56	18.96
64	Val	7.99	119.59	4.07	2.08	0.93, 0.93	-	176.01	62.30	32.63
65	Val	8.26	125.34	4.08	2.03	0.92, 0.92	-	175.81	62.21	32.46
66	Arg	8.49	126.90	4.63	-	-	3.24, 3.24	173.99	53.70	30.05
67	Pro	-	-	4.38	-	-	-	176.75	63.06	32.04
68	His	8.66	118.77	4.64	3.25, 3.25	-	-	174.09	55.26	28.86
69	Ala	8.42	125.50	4.31	1.39	-	-	177.51	52.45	19.25
70	Gln	8.56	120.76	4.36	-	-	-	175.78	55.60	29.44
71	Ile	8.40	124.95	4.47	1.87	-	-	-	58.76	38.55
72	Pro	-	-	4.42	-	-	-	176.76	63.19	32.01
73	Gln	8.54	121.29	4.30	-	-	-	176.11	55.97	29.59
74	Arg	8.46	122.86	4.27	-	-	-	175.84	56.19	30.64
75	Gln	8.47	121.88	4.31	-	-	-	175.27	55.75	29.70
76	Tyr	8.40	122.50	4.61	-	-	-	175.09	57.63	38.87
77	Leu	8.25	126.23	4.62	-	-	-	-	52.55	42.04
78	Pro	-	-	4.33	-	-	-	176.75	63.22	31.90
79	Asn	8.55	118.25	4.65	2.82, 2.82	-	-	175.21	53.24	38.52
80	Ser	8.19	116.03	4.39	3.83, 3.83	-	-	173.88	58.26	63.87
81	His	8.48	120.30	4.99	-	-	-	171.79	53.29	28.47
83	Pro	-	-	4.53	-	-	-	176.95	63.02	31.92
84	Thr	8.39	116.23	4.30	4.11	1.19	-	174.41	61.97	69.72
85	Val	8.32	124.60	4.14	2.03	0.93, 0.93	-	175.76	62.19	32.87
86	Val	8.41	126.34	4.06	1.98	0.93, 0.93	-	175.86	62.29	32.64
87	Arg	8.55	126.62	4.36	-	-	3.20, 3.20	175.80	55.88	30.75
88	Arg	8.53	124.46	4.62	-	-	3.24, 3.24	174.20	53.91	30.16
89	Pro	-	-	4.38	-	-	-	176.35	63.17	32.02
90	Asn	8.59	119.19	4.65	2.78, 2.78	-	-	175.06	52.94	38.48
91	Leu	8.29	123.04	4.28	-	-	-	176.88	55.17	42.38
92	His	8.50	118.83	4.70	3.20, 3.20	-	-	172.04	53.06	28.81
93	Pro	-	-	-	-	-	-	176.85	63.23	32.06
94	Ser	8.51	116.51	4.43	3.84, 3.84	-	-	173.99	58.19	63.85
95	Phe	8.28	122.55	4.67	3.08, 3.08	-	-	174.99	57.73	39.78

96	Ile	8.04	124.41	4.07	-	-	-	174.99	60.51	38.86
97	Ala	8.34	129.13	4.28	1.36	-	-	177.21	52.16	19.13
98	Ile	8.29	123.22	4.45	-	-	-	-	-	-
100	Pro	-	-	4.44	-	-	-	176.91	62.76	32.02
101	Lys	8.40	121.90	4.25	-	-	-	176.61	56.34	33.08
102	Lys	8.43	123.56	4.33	-	-	-	176.40	56.25	33.04
103	Ile	8.34	123.83	4.16	-	-	-	176.15	61.02	38.60
104	Gln	8.59	125.35	4.35	-	-	-	175.44	55.62	29.51
105	Asp	8.60	121.56	4.71	-	-	-	174.75	52.86	38.18
106	Lys	8.36	122.58	4.33	-	-	-	176.01	56.31	32.99
107	Ile	8.24	123.72	4.14	-	-	-	175.88	60.97	38.54
108	Ile	8.44	127.48	4.18	-	-	-	175.90	60.46	38.40
109	Ile	8.47	129.04	4.48	-	-	-	-	-	-
110	Pro	-	-	4.47	-	-	-	176.89	63.18	32.18
111	Thr	8.35	115.62	4.32	4.15	1.23	-	174.78	61.91	69.72
112	Ile	8.33	124.14	4.20	1.89	-	-	176.88	61.33	38.66
113	Gly	8.63	113.43	3.99, 3.99	-	-	-	174.77	45.27	-
114	Gly	8.33	108.76	3.97, 3.97	-	-	-	174.36	45.22	-
115	Ser	8.30	115.13	4.35	3.79, 3.79	-	-	174.78	58.27	63.68
116	His	8.59	119.76	4.67	-	-	-	174.21	55.13	28.33
117	His	8.50	118.71	4.68	3.17, 3.17	-	-	174.16	55.17	28.81
118	His	8.71	119.80	4.70	-	-	-	174.15	55.22	29.03
119	His	8.80	120.48	4.69	-	-	-	173.98	55.29	29.13
120	His	8.77	121.15	4.65	-	-	-	173.87	55.64	29.14
121	His	8.77	122.98	4.67	-	-	-	176.47	55.25	28.82

Table 2 The values of $^3J(H^N\text{-}H^\alpha)$ BME-RL2 at pH 3.5

	$^3J(H^N\text{-}H^\alpha)$, Hz		$^3J(H^N\text{-}H^\alpha)$, Hz
2 Asn	6.8	69 Ala	4.7
3 Gln	7.3	70 Gln	5.7
4 Lys	6.1	71 Ile	7.0
7 Ala	5.6	73 Gln	6.5
8 Cys	6.4	75 Gln	7.9
9 His	7.9	76 Tyr	7.1
11 Asn	6.8	77 Leu	7.5
12 Asp	8.3	79 Asn	7.0
13 Glu	6.8	80 Ser	6.9
17 Tyr	7.1	81 His	7.9
18 Gln	5.8	84 Thr	6.8
20 Thr	7.2	85 Val	9.0
21 Ala	5.0	86 Val	7.5
31 Asn	6.7	87 Arg	7.1
33 Tyr	5.8	88 Arg	6.3
38 Thr	7.8	90 Asn	7.3
39 Asn	6.5	94 Ser	6.9
40 Leu	5.9	95 Phe	5.8

41 Tyr	6.9	96 Ile	8.1
42 Gln	7.6	97 Ala	5.5
43 Arg	5.7	101 Lys	5.4
46 Ala	6.1	102 Lys	6.4
47 Ile	7.5	103 Ile	7.1
48 Ala	5.1	104 Gln	7.1
49 Ile	7.0	105 Asp	6.7
50 Asn	6.9	106 Lys	6.6
51 Asn	7.6	107 Ile	7.2
56 Arg	6.7	108 Ile	8.6
57 Thr	6.9	109 Ile	7.9
58 Tyr	6.8	111 Thr	7.4
59 Tyr	8.0	112 Ile	6.7
60 Ala	5.8	115 Ser	6.4
61 Asn	6.4	116 His	7.9
64 Val	7.8	118 His	7.4
65 Val	7.9	119 His	7.2
66 Arg	6.7	120 His	6.7
68 His	7.0	121 His	7.9

Table 3 Relaxation data and SSP results for BME-RL2 at pH 3.5

	T_1, s	T_2, s	T_1 / T_2	NOE	SSP
1Met	-	-	-	-	-
2Asn	1.190±0.022	0.987±0.056	1.206±0.072	-1.589±0.065	-0.133
3Gln	0.961±0.017	0.734±0.028	1.309±0.056	-0.942±0.050	-0.133
4Lys	0.801±0.015	0.571±0.046	1.403±0.116	-0.569±0.099	-0.161
5Gln	0.793±0.023	0.564±0.076	1.406±0.194	-0.347±0.082	-
6Pro	-	-	-	-	-0.164
7Ala	0.687±0.014	0.347±0.017	1.977±0.103	0.032±0.026	-0.212
8Cys	0.685±0.020	0.551±0.081	1.243±0.187	-0.010±0.073	-
9His	0.655±0.050	0.414±0.054	1.580±0.238	-0.084±0.110	-0.045
10Glu	0.700±0.016	0.468±0.086	1.495±0.277	-0.168±0.069	-0.034
11Asn	0.649±0.013	0.374±0.017	1.737±0.088	-0.138±0.056	0.005
12Asp	0.668±0.019	0.357±0.029	1.871±0.160	0.127±0.044	0.055
13Glu	0.657±0.006	0.294±0.015	2.232±0.116	-0.004±0.019	0.022
14Arg	0.678±0.038	0.390±0.042	1.741±0.210	-	-
15Pro	-	-	-	-	-
16Phe	-	-	-	-	-0.139
17Tyr	0.661±0.022	0.254±0.011	2.606±0.140	-0.120±0.030	-0.106
18Gln	0.609±0.032	0.248±0.021	2.452±0.243	-	-0.027
19Lys	0.670±0.017	0.264±0.012	2.542±0.136	-	-0.026
20Thr	0.685±0.031	0.282±0.022	2.426±0.219	-0.054±0.039	-0.033
21Ala	0.716±0.023	0.263±0.012	2.724±0.148	-0.166±0.056	-
22Pro	-	-	-	-	0.112
23Tyr	0.667±0.016	0.255±0.017	2.620±0.188	-0.029±0.032	-0.088
24Val	0.629±0.018	0.220±0.010	2.858±0.158	0.140±0.048	-
25Pro	-	-	-	-	-0.077
26Met	0.648±0.016	0.232±0.009	2.794±0.123	0.158±0.017	-0.134
27Tyr	0.622±0.014	0.219±0.009	2.837±0.132	0.146±0.033	-0.134
28Tyr	0.620±0.011	0.226±0.009	2.744±0.121	0.199±0.037	-0.059
29Val	0.619±0.023	0.216±0.014	2.861±0.208	0.028±0.032	-
30Pro	-	-	-	-	0.121
31Asn	0.662±0.018	0.242±0.012	2.737±0.155	0.229±0.062	0.197
32Ser	0.667±0.021	0.239±0.009	2.795±0.137	0.067±0.027	0.197
33Tyr	0.623±0.018	0.236±0.010	2.639±0.137	-	-
34Pro	-	-	-	-	0.093
35Tyr	0.640±0.022	0.188±0.006	3.404±0.165	0.257±0.046	0.072
36Tyr	0.611±0.012	0.201±0.006	3.046±0.105	0.237±0.037	0.111
37Gly	0.627±0.017	0.255±0.007	2.458±0.092	0.198±0.040	0.084
38Thr	0.639±0.010	0.259±0.009	2.463±0.090	0.211±0.022	0.174
39Asn	0.632±0.006	0.243±0.007	2.600±0.080	0.219±0.032	0.2
40Leu	0.625±0.016	0.226±0.002	2.768±0.075	0.246±0.020	0.143

41Tyr	0.605±0.009	0.221±0.006	2.735±0.083	0.086±0.026	0.097
42Gln	0.613±0.014	0.236±0.012	2.592±0.145	0.330±0.052	0.11
43Arg	0.616±0.013	0.252±0.015	2.446±0.150	0.155±0.025	-0.051
44Arg	0.627±0.009	0.241±0.010	2.599±0.114	-	-
45Pro	-	-	-	-	-0.153
46Ala	0.680±0.013	0.261±0.007	2.605±0.084	0.066±0.022	-0.189
47Ile	0.709±0.016	0.275±0.007	2.577±0.084	-0.093±0.029	-0.125
48Ala	0.697±0.009	0.258±0.004	2.702±0.056	-0.216±0.029	-0.103
49Ile	0.750±0.011	0.287±0.008	2.615±0.080	0.012±0.020	-0.092
50Asn	0.707±0.013	0.282±0.011	2.512±0.107	0.073±0.020	-0.118
51Asn	0.749±0.021	0.301±0.023	2.483±0.205	-0.137±0.028	-
52Pro	-	-	-	-	0
53Tyr	0.652±0.015	0.250±0.013	2.605±0.147	0.059±0.025	-0.064
54Val	0.679±0.015	0.228±0.005	2.982±0.090	0.029±0.027	-
55Pro	-	-	-	-	0.051
56Arg	0.678±0.017	0.278±0.007	2.440±0.088	0.011±0.023	-0.001
57Thr	0.677±0.013	0.263±0.011	2.578±0.115	0.052±0.022	-0.023
58Tyr	0.668±0.022	0.257±0.012	2.601±0.147	0.015±0.075	-0.023
59Tyr	0.660±0.010	0.255±0.005	2.592±0.067	-0.076±0.019	-0.002
60Ala	0.690±0.016	0.296±0.010	2.329±0.094	-0.062±0.021	-0.116
61Asn	0.754±0.014	0.313±0.007	2.410±0.069	-0.250±0.022	-
62Pro	-	-	-	-	-0.033
63Ala	0.690±0.012	0.303±0.011	2.278±0.093	-0.066±0.053	0.052
64Val	0.732±0.013	0.320±0.012	2.291±0.093	-0.260±0.024	0.052
65Val	0.698±0.010	0.312±0.011	2.236±0.088	-0.269±0.023	0.022
66Arg	0.724±0.016	0.294±0.007	2.463±0.077	-0.172±0.020	-
67Pro	-	-	-	-	-0.055
68His	0.723±0.015	0.305±0.009	2.375±0.087	-0.330±0.021	-0.216
69Ala	0.760±0.014	0.350±0.013	2.170±0.091	-0.283±0.020	-0.216
70Gln	0.793±0.012	0.336±0.008	2.359±0.066	-0.156±0.021	-0.204
71Ile	0.756±0.018	0.325±0.016	2.324±0.131	-0.281±0.025	-
72Pro	-	-	-	-	-0.188
73Gln	0.733±0.024	0.326±0.008	2.248±0.089	-0.164±0.021	-0.184
74Arg	0.720±0.014	0.350±0.010	2.058±0.071	-0.150±0.019	-0.174
75Gln	0.686±0.013	0.310±0.011	2.210±0.089	-0.092±0.020	-0.163
76Tyr	0.696±0.015	0.346±0.016	2.009±0.100	-0.146±0.019	-0.132
77Leu	0.700±0.013	0.301±0.009	2.326±0.085	-0.159±0.019	-
78Pro	-	-	-	-	0.015
79Asn	0.743±0.015	0.343±0.010	2.163±0.078	-0.061±0.020	0.08
80Ser	0.760±0.013	0.401±0.017	1.893±0.087	-0.032±0.021	0.08
81His	0.747±0.014	0.346±0.011	2.163±0.079	-0.170±0.022	-
82Pro	-	-	-	-	-
83Pro	-	-	-	-	0.026

84Thr	0.774±0.015	0.347±0.010	2.230±0.079	-0.413±0.026	0.047
85Val	0.742±0.014	0.317±0.014	2.344±0.110	-0.302±0.020	0.003
86Val	0.752±0.013	0.317±0.005	2.372±0.056	-0.338±0.023	0.046
87Arg	0.736±0.016	0.328±0.004	2.244±0.055	-0.177±0.021	-0.046
88Arg	0.744±0.015	0.296±0.005	2.514±0.066	-0.132±0.019	-
89Pro	-	-	-	-	-0.106
90Asn	0.766±0.014	0.326±0.007	2.346±0.067	-0.303±0.023	-0.034
91Leu	0.732±0.024	0.415±0.026	1.763±0.126	-	-0.074
92His	0.749±0.015	0.344±0.008	2.177±0.066	-0.239±0.057	-
93Pro	-	-	-	-	-0.035
94Ser	0.778±0.011	0.366±0.013	2.124±0.083	-0.198±0.021	-0.096
95Phe	0.744±0.011	0.365±0.011	2.040±0.069	-0.160±0.017	-0.12
96Ile	0.761±0.017	0.333±0.006	2.287±0.066	-0.434±0.024	-0.103
97Ala	0.737±0.015	0.364±0.008	2.028±0.061	-0.306±0.023	-0.164
98Ile	0.757±0.019	0.289±0.020	2.619±0.193	-	-
99Pro	-	-	-	-	-
100Pro	-	-	-	-	-0.261
101Lys	0.723±0.012	0.308±0.011	2.345±0.090	-0.229±0.028	-0.19
102Lys	0.716±0.011	0.321±0.011	2.231±0.082	-0.192±0.016	-0.201
103Ile	0.704±0.007	0.318±0.012	2.217±0.088	-0.328±0.017	-0.151
104Gln	0.730±0.014	0.337±0.004	2.166±0.050	-0.262±0.022	-0.164
105Asp	0.770±0.011	0.381±0.013	2.022±0.073	-0.382±0.023	-0.108
106Lys	0.739±0.018	0.379±0.010	1.951±0.070	-0.359±0.021	-0.125
107Ile	0.706±0.010	0.370±0.011	1.910±0.062	-0.148±0.020	-0.089
108Ile	0.681±0.009	0.356±0.006	1.914±0.042	-0.115±0.023	-0.131
109Ile	0.703±0.011	0.374±0.017	1.881±0.089	-0.086±0.020	-
110Pro	-	-	-	-	0.006
111Thr	0.729±0.014	0.458±0.018	1.592±0.071	-0.419±0.022	0.001
112Ile	0.745±0.015	0.463±0.008	1.608±0.043	-0.481±0.020	-0.026
113Gly	0.747±0.014	0.497±0.014	1.505±0.051	-0.285±0.019	0.094
114Gly	0.826±0.017	0.634±0.018	1.302±0.045	-0.599±0.025	-0.005
115Ser	0.830±0.011	0.668±0.052	1.243±0.098	-0.610±0.024	-0.059
116His	0.786±0.013	0.642±0.022	1.225±0.047	-0.422±0.020	-0.062
117His	0.808±0.017	0.688±0.046	1.175±0.082	-0.300±0.042	-0.06
118His	0.838±0.016	0.706±0.055	1.188±0.096	-0.922±0.033	-0.161
119His	0.879±0.016	0.756±0.051	1.163±0.081	-0.633±0.024	-0.155
120His	0.935±0.016	0.843±0.040	1.109±0.056	-1.015±0.031	-0.141
121His	1.090±0.017	1.022±0.045	1.067±0.050	-1.416±0.043	-0.122

Table 4 Chemical shifts of BME-RL2 at pH=3.9 (Acetate buffer)

N		δH	δN	δH$^{\alpha}$	δH$^{\beta}$	δH$^{\gamma}$	δH$^{\delta}$	δC′	δC$^{\alpha}$	δC$^{\beta}$
1	Met	-	-	-				172.08	55.07	32.81
2	Asn	8.99	122.43	4.80				174.76	53.36	38.84
3	Gln	8.64	121.78	4.34				175.80	55.94	29.62
4	Lys	8.50	123.24	4.30				176.42	56.50	32.96
5	Gln	8.50	123.05	4.62				173.94	53.68	28.92
6	Pro	-	-	-				176.50	63.08	32.10
7	Ala	8.48	124.10	4.31				177.59	52.44	19.23
8	Cys	8.44	118.39	4.64				174.57	55.40	41.12
9	His	8.78	121.19	4.75				174.22	55.40	28.95
10	Glu	8.53	122.27	4.29				175.99	56.57	29.41
11	Asn	8.66	119.67	4.71				174.98	53.41	38.84
12	Asp	8.35	120.52	4.63				175.87	54.19	40.28
13	Glu	8.35	120.66	4.35				176.06	56.14	29.20
14	Arg	8.29	122.79	4.49				174.13	54.40	30.07
15	Pro	-	-	-				176.63	63.17	31.90
16	Phe	8.28	119.99	4.50				175.36	58.04	39.48
17	Tyr	7.91	121.52	4.49				175.11	57.74	38.92
18	Gln	8.13	122.54	4.26				175.41	55.59	29.80
19	Lys	8.35	123.13	4.24				176.68	56.78	33.07
20	Thr	8.11	115.04	4.28				173.80	61.54	69.89
21	Ala	8.34	127.91	4.59				175.36	50.61	18.44
22	Pro	-	-	-				176.31	63.10	31.86
23	Tyr	8.24	120.83	4.53				175.05	57.83	38.82
24	Val	7.91	126.33	4.25				173.31	59.10	33.24
25	Pro	-	-	-				176.42	63.07	31.98
26	Met	8.24	120.02	4.52				175.47	55.60	33.18
27	Tyr	8.03	120.03	-				174.85	57.27	39.05
28	Tyr	-	-	-				174.63	57.73	39.25
29	Val	8.00	124.87	-				173.87	59.36	33.03
30	Pro	-	-	-				176.61	63.26	32.05
31	Asn	8.50	117.93	4.58				174.94	53.69	38.61
32	Ser	8.08	114.75	4.26				173.62	58.15	64.02
33	Tyr	8.17	122.84	4.75				174.13	56.03	38.44
34	Pro	-	-	-				176.06	63.27	31.54
35	Tyr	7.87	119.60	-				175.48	57.85	38.82
36	Tyr	8.08	122.02	4.51				176.05	57.89	38.92
37	Gly	7.78	109.77	3.92				174.05	45.42	-
38	Thr	8.07	112.78	4.36				174.48	61.83	69.87
39	Asn	8.53	120.60	4.68				175.25	53.45	38.62
40	Leu	8.13	121.97	4.19				177.13	55.82	42.27
41	Tyr	8.05	119.25	4.50				175.67	57.98	38.46
42	Gln	8.06	121.19	4.26				175.41	55.74	29.58

43	Arg	8.28	122.11	4.29				175.93	56.13	30.78
44	Arg	8.38	123.59	4.57				173.99	53.94	30.28
45	Pro	-	-	-				176.42	63.04	32.12
46	Ala	8.44	124.60	4.28				177.66	52.54	19.25
47	Ile	8.07	119.84	4.14				175.72	60.81	38.98
48	Ala	8.39	128.45	4.37				177.41	52.33	19.35
49	Ile	8.16	120.34	4.15				175.85	61.12	38.97
50	Asn	8.51	122.29	4.68				174.27	53.11	38.99
51	Asn	8.39	120.53	4.95				173.32	51.25	38.96
52	Pro	-	-	-				176.46	63.25	31.97
53	Tyr	8.25	120.39	4.58				175.25	58.00	38.46
54	Val	7.76	125.12	4.31				173.53	59.31	33.10
55	Pro	-	-	-				176.76	62.95	32.09
56	Arg	8.44	121.41	4.31				176.38	56.24	30.91
57	Thr	8.08	115.01	4.26				173.81	61.70	69.83
58	Tyr	8.24	122.92	4.51				174.97	58.00	39.22
59	Tyr	8.06	122.40	4.47				174.80	57.50	39.21
60	Ala	8.12	125.87	4.23				176.63	52.01	19.46
61	Asn	8.34	119.34	4.88				173.62	51.14	38.86
62	Pro	-	-	-				176.56	63.33	32.11
63	Ala	8.26	123.12	4.27				177.81	52.55	19.15
64	Val	8.00	119.53	4.08				176.01	62.33	32.75
65	Val	8.27	125.26	4.09				175.80	62.15	32.58
66	Arg	8.50	126.88	4.64				174.03	53.77	30.23
67	Pro	-	-	-				176.77	62.99	32.13
68	His	8.67	118.77	4.65				174.11	55.20	29.00
69	Ala	8.43	125.47	4.32				177.51	52.44	19.43
70	Gln	8.57	120.73	4.35				175.78	55.64	29.56
71	Ile	8.41	124.87	4.48				174.66	58.76	38.58
72	Pro	-	-	-				176.77	63.23	32.15
73	Gln	8.56	121.28	4.29				176.13	55.85	29.61
74	Arg	8.46	122.79	4.27				175.84	56.29	30.80
75	Gln	8.48	121.82	4.31				175.28	55.68	29.74
76	Tyr	8.41	122.43	4.60				175.12	57.57	38.97
77	Leu	8.26	126.16	4.62				174.92	52.67	42.03
78	Pro	-	-	-				176.77	63.25	31.93
79	Asn	8.57	118.22	4.66				175.23	53.24	38.61
80	Ser	8.20	115.99	4.40				173.88	58.31	63.82
81	His	8.49	120.33	4.98				171.82	53.33	28.58
83	Pro	-	-	-				176.96	62.97	32.01
84	Thr	8.40	116.19	4.30				174.42	62.15	69.79
85	Val	8.33	124.49	4.15				175.79	62.08	32.92
86	Val	8.42	126.28	4.08				175.88	62.24	32.73
87	Arg	8.57	126.59	4.35				175.80	55.72	30.85
88	Arg	8.54	124.45	4.61				174.24	53.94	30.15
89	Pro	-	-	-				176.38	63.10	32.14

90	Asn	8.60	119.16	4.64				175.09	52.94	38.52
91	Leu	8.30	123.02	4.27				176.88	55.22	42.39
92	His	8.51	118.80	4.98				172.10	53.09	29.01
93	Pro	-	-	-				176.88	63.18	32.28
94	Ser	8.52	116.47	4.43				174.00	58.27	63.80
95	Phe	8.29	122.46	4.69				175.03	57.60	39.90
96	Ile	8.05	124.28	4.07				175.02	60.53	38.89
97	Ala	8.35	129.09	4.29				177.20	52.13	19.20
98	Ile	8.29	123.13	4.44				174.40	58.42	38.68
100	Pro	-	-	-				176.90	62.73	32.16
101	Lys	8.41	121.93	4.25				176.61	56.31	33.23
102	Lys	8.46	123.67	4.33				176.48	56.27	33.15
103	Ile	8.36	123.80	4.16				176.22	61.07	38.66
104	Gln	8.59	125.17	4.34				175.50	55.86	29.54
105	Asp	8.47	122.53	4.59				175.71	54.22	40.56
106	Lys	8.33	121.75	4.32				176.15	56.26	33.11
107	Ile	8.26	123.43	4.14				175.91	61.06	38.50
108	Ile	8.44	127.29	4.19				175.91	60.43	38.41
109	Ile	8.48	128.91	4.49				174.59	58.24	38.34
110	Pro	-	-	-				176.92	63.17	32.22
111	Thr	8.35	115.53	4.33				174.82	61.86	69.83
112	Ile	8.34	124.06	4.21				176.87	61.26	38.71
113	Gly	8.64	113.48	4.00				174.79	45.37	-
114	Gly	8.34	108.81	3.98				174.38	45.31	-
115	Ser	8.32	115.21	4.37				174.79	58.42	63.65
116	His	8.61	119.86	4.67				174.22	55.22	28.49
117	His	8.51	118.88	4.66				174.20	55.26	28.83
118	His	8.73	120.00	4.70				174.21	55.28	29.19
119	His	8.81	120.71	4.70				174.01	55.41	29.12
120	His	8.67	120.87	4.66				173.57	55.55	29.33
121	His	8.50	125.78	4.46				178.95	57.24	29.86

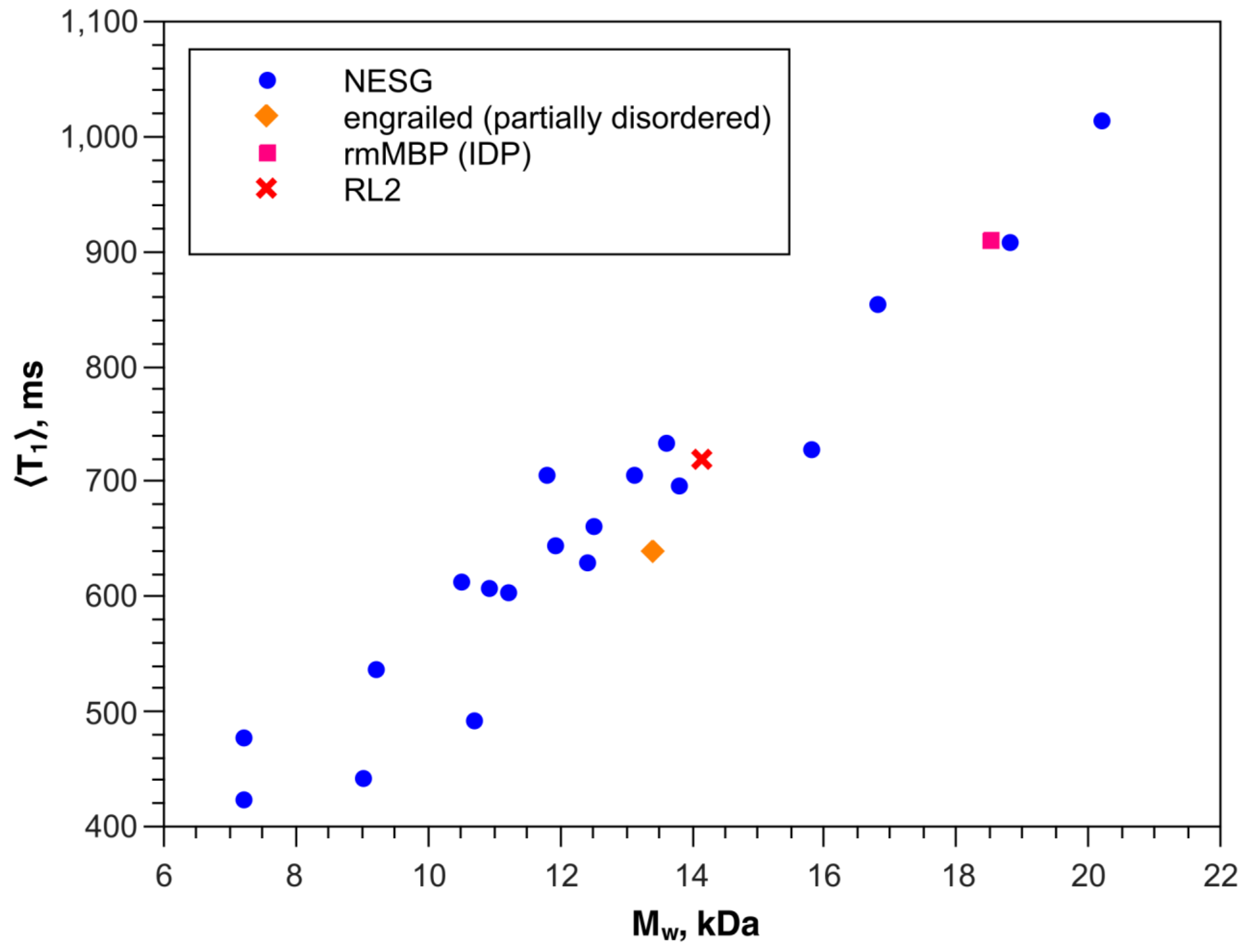

Fig. S6. Comparison of protein <T_1> values

1) **NESG targets**, 600MHz, 298K,
http://www.nmr2.buffalo.edu/nesg.wiki/NMR_determined_Rotational_correlation_time

2) **engrailed**, 600MHz, 303K, <T_1> = 0.64s, M_w = 13.4 kDa. Biophysical Journal 109(5) 988–999. DOI: 10.1016/j.bpj.2015.06.069 (Calculated from SI data)

3) **rmMBP**, 600MHz, 300K, <T_1> = 0.91s, M_w = 18.5 kDa. Biophysical Journal 94(12) 4847–4866. DOI: 10.1529/biophysj.107.125823

4) **RL2**, 600MHz, 293.2K, <T_1> = 0.72s, M_w = 14.14 kDa.

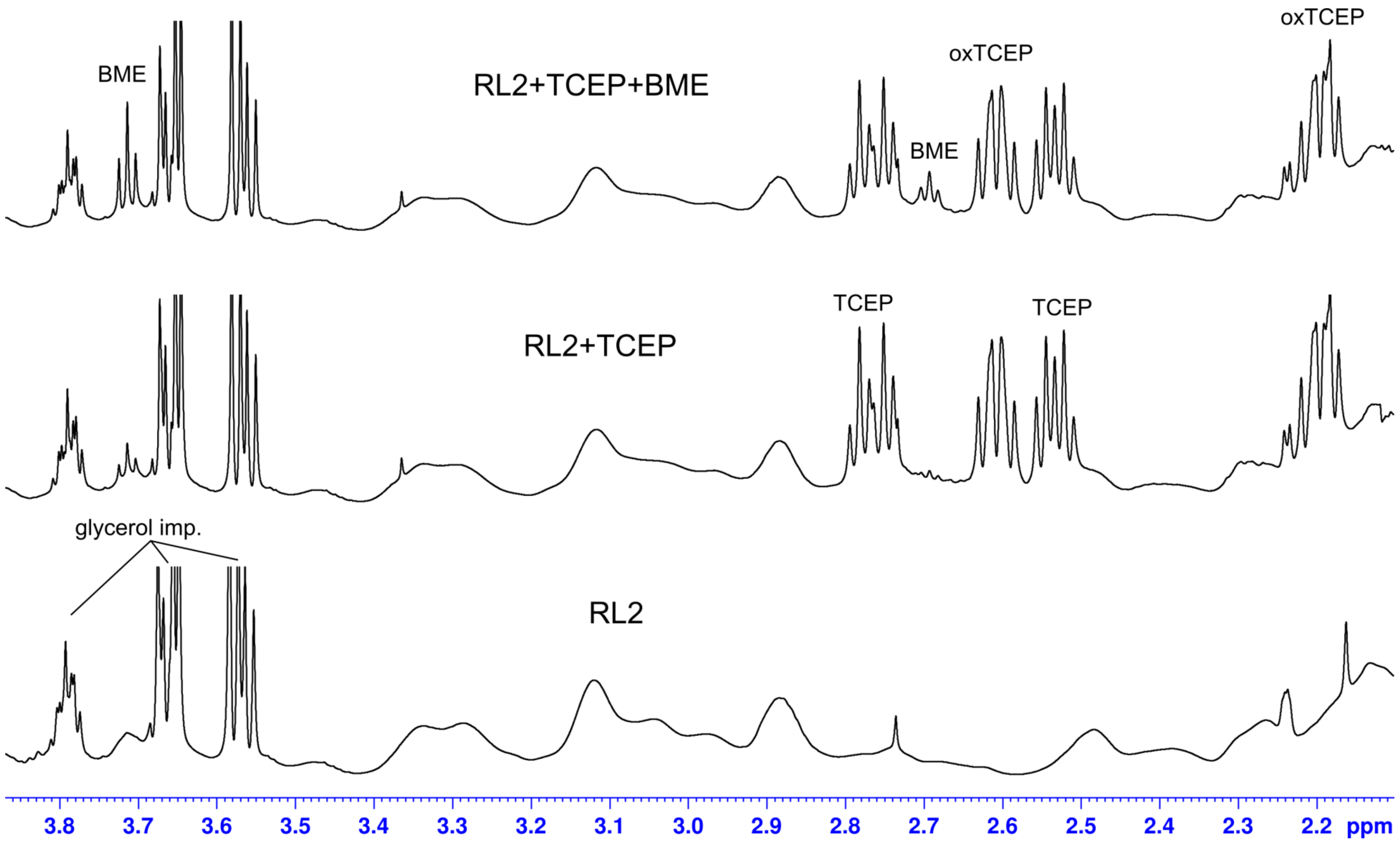

Fig. S7. Fragment of ^{1}H NMR spectrum of [U-^{13}C,^{15}N]-RL2 (acetate buffer, pH 3.9): before (bottom) and after TCEP addition (middle), with external additive BME (top).

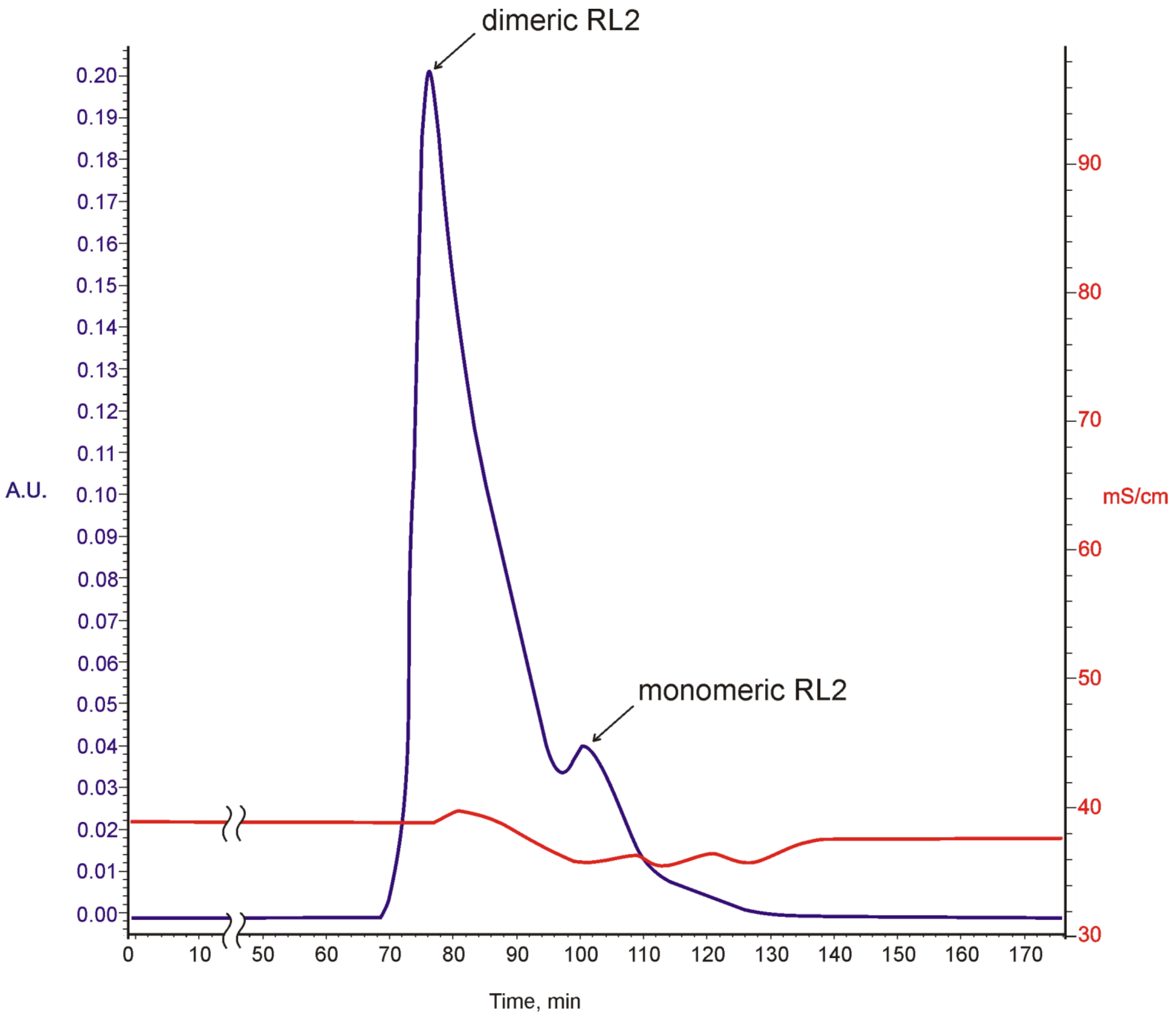

Fig. S8. Gel filtration chromatograms showing the separation of RL2 dimer from the monomer.
